# Walnut Polyphenol Extract Protects against Malathion- and Chlorpyrifos-Induced Immunotoxicity by Modulating TLRx-NOX-ROS

**DOI:** 10.3390/nu12030616

**Published:** 2020-02-27

**Authors:** Yue Zhao, Chang Fan, Ao Zhang, Yue Zhang, Fengjun Wang, Qiang Weng, Meiyu Xu

**Affiliations:** 1 Collage of Biological Science and Technology, Beijing Forestry University, Beijing 100083, China; zhaoyue7180171@bjfu.edu.cn (Y.Z.); fanchang@bjfu.edu.cn (C.F.); zhangaoo@bjfu.edu.cn (A.Z.); zy15810252987@bjfu.edu.cn (Y.Z.); wangfengjun@bjfu.edu.cn (F.W.); qiangweng@bjfu.edu.cn (Q.W.); 2 Beijing Key Laboratory of Forest Food Processing and Safety, Beijing Forestry University, Beijing 100083, China

**Keywords:** walnut polyphenol extract, malathion, chlorpyrifos, immunotoxicity, splenic lymphocytes

## Abstract

Malathion (MT) and chlorpyrifos (CPF) are immunotoxic organophosphate pesticides that are used extensively in agriculture worldwide. Dietary polyphenols protect against a variety of toxins. In this study, walnut polyphenol extract (WPE) prevents MT- or CPF-induced toxicity to splenic lymphocytes in vitro. WPE promotes the proliferation of MT-exposed splenocytes, as indicated by increases in the proportions of splenic T-lymphocyte subpopulations (CD3^+^, CD4^+^, and CD8^+^ T cells) and levels of T-cell-related cytokines interleukin (IL)-2, interferon-γ, IL-4, and granzyme B, and decreases the apoptosis-associated proteins Bax and p53. WPE also significantly enhances the proliferation of CPF-exposed splenic B lymphocytes (CD19^+^ B cells) and levels of the B-cell-related cytokine IL-6, leading to decreases of the apoptosis-associated proteins Bax and p53. These effects are related to reduced production of reactive oxygen species (ROS), as evidenced by normalized hydroxyl radical (•OH), malondialdehyde (MDA), superoxide dismutase (SOD), glutathione peroxidase (GSH-Px), catalase (CAT), and glutathione (GSH) levels, which are associated with decreased expression of NADPH oxidase 2 (NOX2) and dual oxidase 1 (DUOX1). WPE inhibits the production of ROS and expression of NOX by regulating toll-like receptors 4 and 7 in MT- and CPF-exposed splenic lymphocytes. In conclusion, WPE protects against MT- or CPF-mediated immunotoxicity and inhibits oxidative damage by modulating toll-like receptor (TLR)x-NOX-ROS.

## 1. Introduction

Malathion (MT) O,O-dimethyl-S-1,2-dicarboethoxy-ethyl-dithiophosphate, one of the most widely used organophosphate pesticides, is distributed extensively in the environment. MT has toxic effects in a variety of tissues in humans and other mammals [1]. Chlorpyrifos (CPF) O,O-diethyl-O-(3,5,6-trichloro-2-pyridyl)-phosphorothioate is an organophosphate pesticide used in agriculture that causes neurotoxicity, reproductive toxicity, respiratory toxicity, hepatotoxicity, and oxidative stress [2,3]. MT and CPF induce immunotoxicity in chicks and rats [3,4,5]. MT alters populations of CD4^+^ and CD8^+^ T lymphocytes and the production of interferon (IFN)-γ and interleukin (IL)-4 in rat spleen [3]. In addition, CPF suppresses splenic cell proliferation and cytokine production in rats and reduces IFN-γ, tumor necrosis factor (TNF)-α, and interleukin (IL)-6 levels in mice [5]. It is important to note that MT and CPF induce overproduction of reactive oxygen species (ROS), resulting in oxidative stress [6,7].

ROS are produced by NADPH oxidase complexes during oxidative phosphorylation [8]. The dominant ROS-generating complex of NADPH oxidase complexes is NADPH oxidase 2 (NOX2) and its homologue dual oxidase 1 (DUOX1) [9,10], which is expressed in, for instance, macrophages, neutrophils, dendritic cells [11], and splenic lymphocytes [12]. Activation of toll-like receptor 4 (TLR4) promotes the interaction of p67 and NOX2, leading to the overproduction of ROS in mononuclear macrophages and primary human monocytes [13]. In neutrophils, lipopolysaccharide (TLR4 agonist) and resiquimod (TLR7 agonist) activate NADPH oxidase, increasing ROS production [14].

Polyphenols possess antioxidant activity, scavenge free radicals [15,16], and protect against multiple toxins. *Ecklonia cava* polyphenol suppresses ethanol-induced death of hepatocytes by scavenging ROS and restoring glutathione (GSH) [17]. A phenolic compound from *Artocarpus heterophyllus* has an antioxidant effect by inhibiting lipopolysaccharide (LPS)-induced ROS generation [18]. Treatment of *Pennisetum glaucum* seeds with polyphenols maintains the functionality of T cells in rat [19]. Similarly, curcumin modulates levels of IFN-γ and IL-1β in T-helper (Th)1 cells in vivo, which prevents nicotine-induced toxicity in rats with protein malnutrition [20]. Immune cells are activated, and their activity is modulated by cytokines [21], and dietary polyphenols influence cytokine production by immune/non-immune cells [20,21,22,23]. Curcumin, a natural polyphenol, modulates the expression of IFN-α, TNF-γ, IL-17, and IL-10 and the proportions of Th subsets, which ameliorate experimental autoimmune myasthenia gravis in rat [21]. Similarly, lychee fruit polyphenols have an anti-inflammatory effect by decreasing the production of proinflammatory cytokines (including IL-6 and TNF-α) by peripheral-blood monocytes [23]. Moreover, polyphenols from red grapes promote immunity in sea bass exposed to microbes and environmental stress by increasing IFN-γ.

Walnut is an important source of polyphenols [24]. Walnut polyphenols protect against 4-pentylphenol- and 3-methyl-4-nitrophenol-induced immunotoxicity, acute lung toxicity induced by cigarette smoke extract, and cisplatin-induced disruptions in motor and cognitive functions in rats and carbon tetrachloride-mediated liver injury in mice [25,26,27]. The protective effects of walnut polyphenols are due at least in part to the attenuation of oxidative damage. The protective effects of walnut polyphenols in fenitrothion-exposed splenic T cells are attributable in part to attenuation of oxidative damage through suppression of the activation of NOX2 and DUOX1. The latter is caused by a reduction in the expression of TLR4 [28]. In our previous research, we found that the average total polyphenol content of walnut polyphenol extract (WPE) was 76.30 ± 1.21 mg gallic acid equivalents (GAE)/g. A total of 19 phenolic compounds was tentatively identified in the WPE, including ellagitannins, gallic tannins, ellagic acid, quercetin, etc. [28]. However, the effects of walnut polyphenols on MT- and CPF-induced immunotoxicity are unclear.

Since MT and CPF induce oxidative damage [7], we evaluated the effects of MT and CPF on splenic lymphocytes in terms of toxicity and oxidative stress. We also assessed the protective effects of walnut polyphenols on MT- and CPF-induced immunotoxicity and the underlying mechanisms.

## 2. Materials and Methods

### 2.1. Materials

Walnuts (Juglans regia L. XiangLing walnut) were obtained from the Jingpin Fruit Industry (Hebei, China). Ellagic acid, proanthocyanidin (grape seed proanthocyanidin) and quercetin were purchased from Shanghai yuanye Bio-Technology (Shanghai, China). MT and CPF were purchased from Balingwei Technology (Shanghai, China). RPMI 1640 medium was purchased from Mediatech (Manassas, VA, USA). Enzyme-linked immunosorbent assay (ELISA) kits of mouse IL-2, IL-4, IL-6, IFN-γ, and granzyme B were purchased from Huamei Biotech (Wuhan, China). Fluorescein isothiocyanate (FITC)-labeled rat IgG2a and IgG2b (negative isotype controls) were obtained from Bio Legend (San Diego, CA, USA). FITC-labeled anti-mouse CD3+(lgG2b), FITC-labeled anti-mouse CD8+(lgG2b), FITC-labeled anti-mouse CD4+(lgG2b), and FITC-labeled anti-mouse CD19+(lgG2a) were also purchased from Bio Legend. Assay kits for superoxide dismutase (SOD), glutathione peroxidase (GSH-Px), hydroxyl radical (•OH), malondialdehyde (MDA), glutathione (GSH), and catalase (CAT) were obtained from Nanjing Jiancheng Bioengineering Institute (Nanjing, China). The following antibodies purchased from Bioss Biotechnology (Beijing, China) were used in the Western blotting studies: rabbit anti-NOX2 antibody, rabbit anti-DUOX1 antibody, rabbit anti-TLR4 antibody, rabbit anti-Bax antibody, rabbit anti-Bcl-2 antibody, rabbit anti-p53 antibody, and secondary antibody (horseradish peroxidase (HRP)-linked anti-rabbit IgG). All other chemicals used here, such as NH4Cl, concanavalin A (Con A), and LPS, were purchased from Sigma (St. Louis, MO, USA).

### 2.2. Experimental Animals

Male Kunming mice (8 weeks old, 20.0 ± 2.0 g weight) were purchased from Peking University Medical Department (Beijing, China). All mice were raised in a pathogen-free facility with a standard 12 h light/dark cycle, which was controlled at 23–25 °C and 57–60% relative humidity. All mice had ad libitum access to standard sterilized rodent chow and filtered water. All procedures were reviewed and approved by the Policy on the Care and Use of Animals established by the Ethics Committee of Beijing Forestry University, which is fully accredited by the Department of Agriculture of Hebei Province, China (JNZF11/2007).

### 2.3. Preparation of Splenocytes

Splenocytes were prepared following the protocols of Yang et al. [27]. Briefly, the mice were euthanized by cervical dislocation and their spleens were removed. Single cell suspensions were prepared in RPMI 1640 (supplemented with 10% fetal bovine serum, 100 U penicillin/mL, 100 mg streptomycin/mL, and 2 mM L-glutamine). In this study, splenocytes stimulated with Con A (5 μg/mL) or LPS (10 μg/mL) were used to investigate the proliferation of splenic T or B cells.

### 2.4. Preparation of the WPE

The WPE was extracted as previously described [27]. Briefly, walnuts (30 g) were stored at –20 °C; the shelled kernels were ground and then immersed in acetate buffer (100 mM, 240 mL), pH 4.8/acetone (30:70, v/v) for 24 h at 4 °C and this process was repeated. The extracts were concentrated using a rotary evaporator until the organic solvent was completely evaporated. The concentrated solution was extracted three times with 75 mL ethyl acetate, and then evaporated to remove the ethyl acetate, and lyophilized; the powder was the WPE.

### 2.5. Cell Viability Assay

Cell viability was measured as previously described [29]. Briefly, 100 μL of splenocyte suspension (5 × 10^6^ cells /mL) were seeded into 96-well culture plates. After 4 h of incubation, designated wells were treated with 100 µL MT or CPF (10^−7^–10^−4^ M) alone or in combination with different concentrations (1, 5, and 10 µg/mL) of WPE. Cells treated with complete medium were used as controls. After another 48 h incubation at 37 °C, 20 µL of 3-(4,5-dimethylthiazol-2-yl)-2,5-diphenyltetrazolium bromide (MTT) (5 mg/mL) solution were added to each well and the plates were incubated for another 4 h. The culture supernatant was removed and 200 µL of DMSO were added to each well. The absorbance (A) was measured at 570 nm using a Bio–Rad Microplate Reader (Hercules, CA, USA).

### 2.6. Flow Cytometry

Lymphocyte phenotypes were analyzed by flow cytometry, as described previously [30]. In short, the splenocytes were washed and diluted to 2.5 × 10^7^ cells/mL in PBS. The cells were stained with 1 µg/mL specific FITC-labeled antibody against CD3, CD4, CD8, and CD19, and then stored in the dark at 4 °C for 30 min. After washing with PBS three times, the splenocytes were transferred to fluorescence-activated cell sorting (FACS) tubes to measure the CD3+, CD4+, and CD8+ T cells and CD19+ B cell subset levels by lymphocyte phenotype analysis. The results were determined using a Becton Dickinson FACSCalibur flow cytometer (San Diego, CA, USA). The results were calculated as the percentage of positive cells recorded by the flow cytometer within a gate that both exposed and controlled splenocytes. All data were analyzed with Emerald Biotech FlowJo software (Hangzhou, China).

### 2.7. ELISA

Splenocytes were incubated with the test reagents (at a density of 5 × 10^6^ cells/mL medium) for 48 h in 96-well plates. The IL-2, IL-4, IL-6, IFN-γ, and granzyme-B levels were measured using commercial ELISA kits.

### 2.8. Antioxidant Enzyme Activities and Biomarkers of Oxidative Stress

To assess the antioxidant enzyme activities and biomarkers of oxidative stress, splenocytes were treated with the test reagents for 48 h at a density of 5 × 10^6^ cells/mL in 96-well plates. Culture supernatants were collected and the GSH-Px, SOD, and CAT activities and GSH, MDA, and •OH levels were determined using commercial assay kits. The lower detection limits of the kits were 0.5 U GSH-Px/mL, 0.5 U SOD/mL, 0.04 U CAT/mL, 0.04 U • OH/mL, 0.01 mmol GSH/mL, and 0.01 mmol MDA/mL, respectively.

### 2.9. Western Blotting

Western blotting was performed as previously described in Liu et al. [28]. Cells were harvested and washed with PBS. Proteins were extracted with radio immunoprecipitation assay (RIPA) lysis buffer containing 10 mg/mL phenylmethanesulfonyl fluoride (PMSF) on ice for 30 min. The protein content was quantified and resolved on SDS-PAGE (10–12% gels) and transferred to polyvinylidene fluoride (PVDF) membranes (Bio-Rad, Richmond, CA, USA). The membranes were blocked with 3% bovine serum albumin (BSA) for 1 h at room temperature and incubated with primary antibodies against NOX2, DUOX1, TLR4, and TLR7 at 4 °C, overnight. Following washing with Tris-buffered saline and Tween20 (TBST) three times, the membranes were incubated with HRP-conjugated secondary antibody at room temperature for 1 h. Finally, the membranes were colored with 10 mg 3,3-diaminobenzidine (DAB) solution in 50 mL phosphate buffer (0.03 M) plus 20 µL H_2_O_2_. β-Actin was used as an endogenous control. Bands were analyzed using Bio-Rad Laboratories ver. 4.5 Quantity One software (Hercules, CA, USA).

### 2.10. Statistical Analysis

All data are expressed as the mean ± SD obtained from at least three individual experiments. Statistical analyses were performed using one-way analysis of variance (ANOVA) followed by a post hoc Tukey’s test with GraphPad Prism software (Version 6.01, GraphPad, San Diego, CA, USA). *p*-values < 0.05 and <0.01 were accepted as significant and very significant, respectively. 

## 3. Results

### 3.1. Effects of WPE on MT- and CPF-Induced Toxicity in Splenocytes

**Figure 1:**
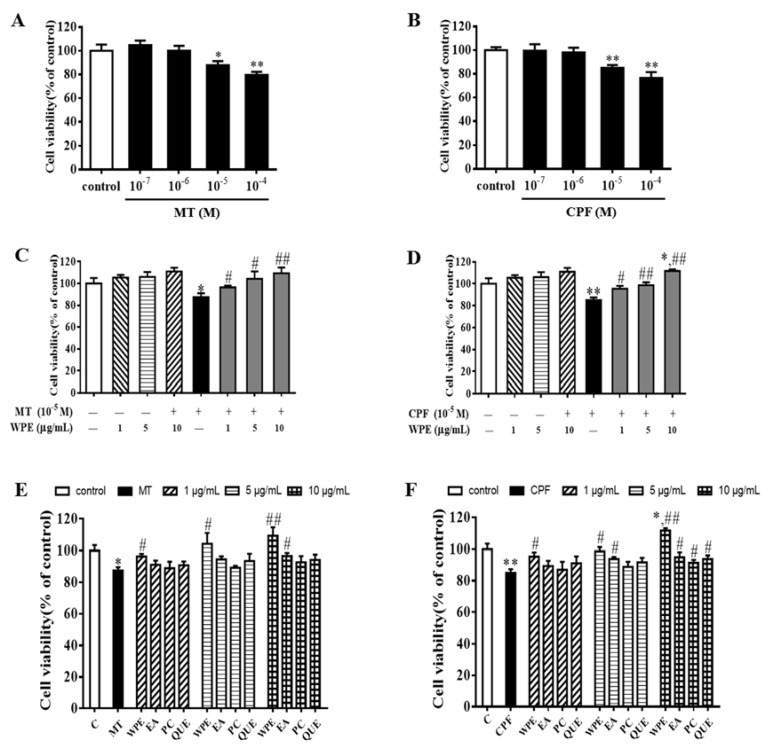
Effect of walnut polyphenol extract (WPE) on cytotoxicity in splenocytes exposed to malathion/chlorpyrifos (MT/CPF). Splenocytes were treated with (**A**) MT (10^−7^–10^−4^ M); (**B**) CPF (10^−7^–10^−4^ M); (**C**) WPE ((1, 5, and 10 µg/mL) or MT (10^−5^ M) or different concentrations (1, 5, and 10 µg/mL) of WPE together with MT; (**D**) WPE (1, 5, and 10 µg/mL) or CPF (10^−5^ M) or different concentrations (1, 5, and 10 µg/mL) of WPE with CPF; (**E**) MT (10^−5^ M) or different concentrations (1, 5, and 10 µg/mL) of WPE or ellagic acid or proanthocyanidin or quercetin with MT; (**F**) CPF (10^−5^ M) or different concentrations (1, 5, and 10 µg/mL) of WPE or ellagic acid or proanthocyanidin or quercetin with CPF. Cell viability was evaluated by a 3-(4,5-dimethylthiazol-2-yl)-2,5-diphenyltetrazolium bromide (MTT) assay. EA, ellagic acid; PC, proanthocyanidin; QUE, quercetin. Results are presented as mean ± SD of three separate experiments. * *p* < 0.05 or ** *p* < 0.01 vs. untreated control; ^#^ *p* < 0.05 or ^##^ *p* < 0.01 vs. MT or CPF treatment.

Splenocytes exposed to MT or CPF were subjected to 3-(4,5-dimethylthiazol-2-yl)-2,5-diphenyltetrazolium bromide (MTT) assay to assess the effects of WPE on their viability. MT and CPF (10^−5^ M) significantly decreased cell viability to 87.8% and 86.1% that of the control (*p* < 0.05; Figure 1A,B). However, WPE at 1~10 µg/mL ameliorated this cytotoxicity in a concentration-dependent manner (Figure 1C,D). Treatment of splenocytes with WPE (1, 5, or 10 µg/mL) increased their viability from 87.5% to 96.4%, 104.3%, and 109.3% for cells treated with 10^-5^ M MT and from 87.8% to 95.4%, 98.7%, and 111.7% for cells treated with 10^-5^ M CPF, respectively, relative to the controls (Figure 1C,D). Ellagic acid, proanthocyanidin, or quercetin increased cell viability, but to a lesser degree than WPE (Figure 1E,F). WPE alone did not affect cell viability, which indicates that it is non-cytotoxic to splenocytes (Figure 1C,D). Therefore, WPE protects against MT- and CPF-induced cytotoxicity in murine splenocytes.

### 3.2. Effects of WPE on the Cytotoxicity of MT and CPF on Splenic Lymphocyte Subpopulations

Treatment with an appropriate concentration of Con A and LPS induced the proliferation of T and B cells, respectively [31,32]. We used splenocytes stimulated by Con A and LPS as splenic T and B cells. To investigate the effects of WPE on splenic T- and B-cell populations exposed to MT or CPF, we treated splenocytes with MT or CPF with or without WPE for 48 h in the presence of Con A or LPS and evaluated their viability by MTT assay.

**Figure 2:**
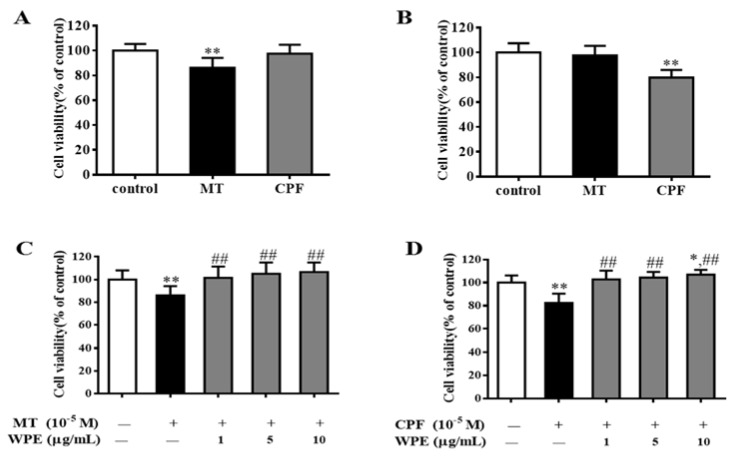
Effect of WPE on cytotoxicity of MT or CPF in splenic lymphocytes subpopulations. Splenocytes were treated with (**A**) MT (10^−5^ M) or CPF (10^−5^ M) in the presence of concanavalin A (Con A) (5 µg/mL, T lymphocytes), control was also stimulated with Con A; (**B**) MT (10^−5^ M) or CPF (10^−5^ M) in the presence of lipopolysaccharide (LPS) (10 µg/mL, B lymphocytes), control was also stimulated with LPS; (**C**) MT (10^−5^ M) alone or different concentrations (1, 5, and 10 µg/mL) of WPE together with MT in the presence of Con A (T lymphocytes); (**D**) CPF (10^−5^ M) alone or different concentrations (1, 5, and 10 µg/mL) of WPE together with CPF in the presence of LPS (B lymphocytes). Results are presented as mean ± SD of three separate experiments. * *p* < 0.05 or ** *p* < 0.01 vs. untreated control; ^#^ *p* < 0.05 or ^##^ *p* < 0.01 vs. MT or CPF treatment.

**Figure 3:**
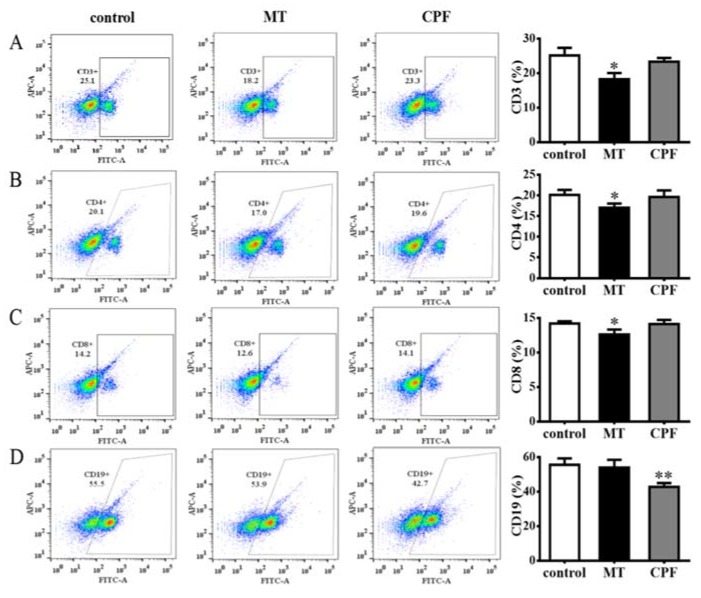
Percentages of various lymphocyte cell types as determined using flow cytometric analysis. (**A**) CD3^+^ T cells, (**B**) CD4^+^ T cells, (**C**) CD8^+^ T cells, and (**D**) CD19^+^ B cells in splenocytes exposed to medium only (control), MT, or CPF. Results are presented as mean ± SD of three separate experiments. * *p* < 0.05 vs. untreated control. ** *p* < 0.01 vs. untreated control.

**Figure 4:**
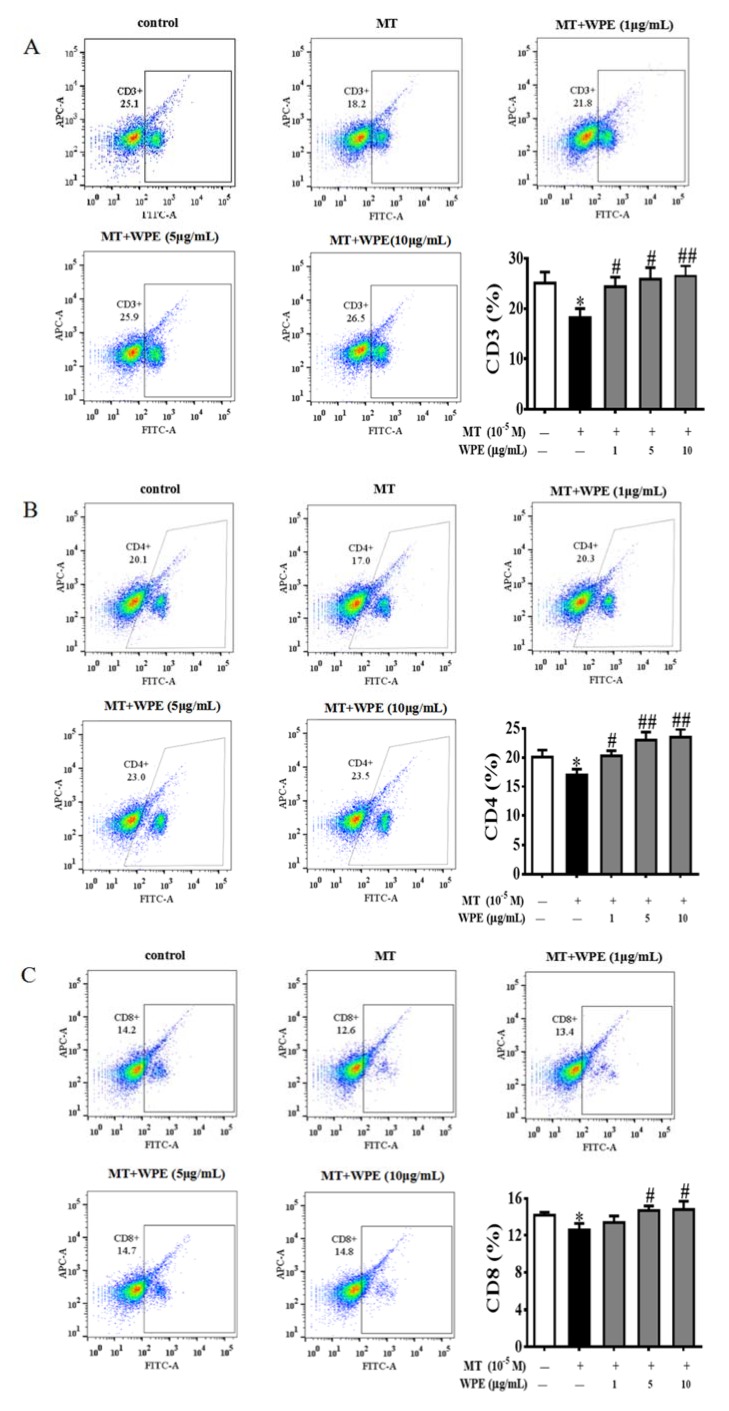

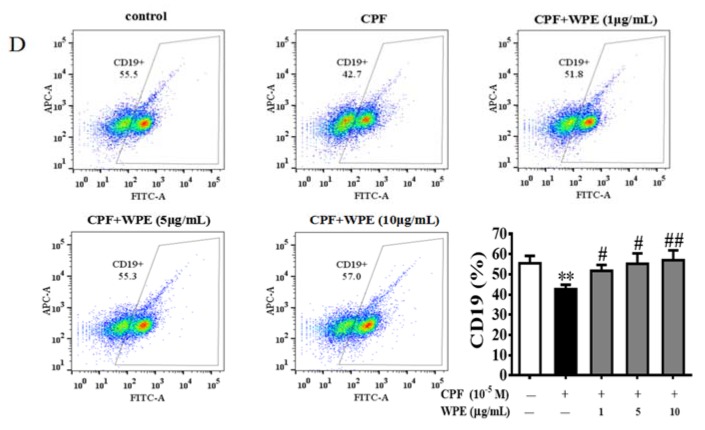
Effect of WPE on MT- and CPF-induced cytotoxicity in splenic lymphocyte subpopulations by flow cytometry. (**A**) CD3^+^ T cells, (**B**) CD4^+^ T cells, (**C**) CD8^+^ T cells, and (**D**) CD19^+^ B cells in splenocytes exposed to medium only (control), MT (10^−5^ M), CPF (10^−5^ M), and WPE (1, 5, and 10 µg/mL) together with MT or CPF. Results are presented as mean ± SD of three separate experiments. * *p* < 0.05 or ** *p* < 0.01 vs. untreated control. ^#^ *p* < 0.05 or ^##^ *p* < 0.01 vs. MT or CPF treatment.

As shown in Figure 2A,B, MT significantly inhibited the proliferation of splenic T cells but not that of splenic B cells, whereas CPF significantly inhibited the proliferation of splenic B cells but not that of splenic T cells. However, WPE (1 µg/mL) significantly attenuated the effects of MT on splenic T cells and the effects of CPF on B cells; the viability of splenic T cells exposed to MT increased from 86.2% to 101.4%, and that of splenic B cells exposed to CPF increased from 82.4% to 102.8% (Figure 2C,D). To further investigate the effects of WPE on splenic lymphocyte subsets exposed to MT or CPF, we reacted splenocytes with fluorescein isothiocyanate (FITC)-labeled antibodies and assessed the results with flow cytometry. As shown in Figure 3, proportions of CD3^+^, CD4^+^, and CD8^+^ T cells were significantly reduced in splenocytes exposed to MT relative to the control; a similar result was obtained for CD19^+^ B cells exposed to CPF. However, MT did not affect the number of CD19^+^ B cells, and CPF did not decrease the number of CD3^+^, CD4^+^, or CD8^+^ T cells, consistent with the above results. WPE significantly increased the proportion of CD3^+^, CD4^+^, and CD8^+^ T cells exposed to MT and the proportion of CD19^+^ B cells exposed to CPF (Figure 4A–D). At 1 µg/mL WPE, the CD3^+^ T-, CD4^+^ T-, and CD19^+^ B-cell populations were similar to the controls (Figure 4A–D). Therefore, WPE is capable of normalizing proportions of splenic T cells exposed to MT or splenic B cells exposed to CPF.

### 3.3. Effects of WPE on Cytokine Production by MT- or CPF-Stimulated Cells

**Figure 5:**
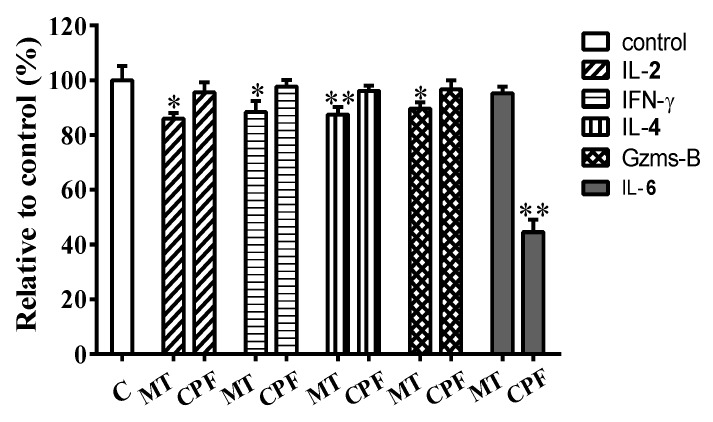
Effect of MT and CPF on cytokine/granzyme production in splenocytes. Splenocytes were cultured for 48 h in the presence of MT (10^−5^ M) or CPF (10^−5^ M). Levels of interleukin (IL)-2, interferon (IFN)-γ, IL-4, granzyme B, and IL-6 released into culture media were then measured by Enzyme-linked immunosorbent assay (ELISA). Results are presented as mean ± SD of three separate experiments. * *p* < 0.05 or ** *p* < 0.01 vs. untreated control.

To investigate the effects of WPE on their function, we treated splenic lymphocytes with MT or CPF in the presence or absence of WPE and analyzed cytokine/granzyme production by enzyme-linked immunosorbent assay (ELISA). IL-2 and IFN-γ were used as markers of CD4^+^ Th1 cells, IL-4 as a marker of CD4^+^ Th2 cells, granzyme B as a marker of CD8^+^ T cells, and IL-6 as a marker of B cells. MT significantly suppressed the synthesis of IL-2, IFN-γ, IL-4, and granzyme B but did not affect that of IL-6 (Figure 5). By contrast, CPF significantly decreased the production of IL-6 but did not affect that of IL-2, IFN-γ, IL-4, or granzyme B (Figure 5). These results, which are consistent with those for cell viability and flow cytometry, show that MT selectively induces toxicity in T cells and CPF selectively induces toxicity in B cells. 

**Figure 6:**
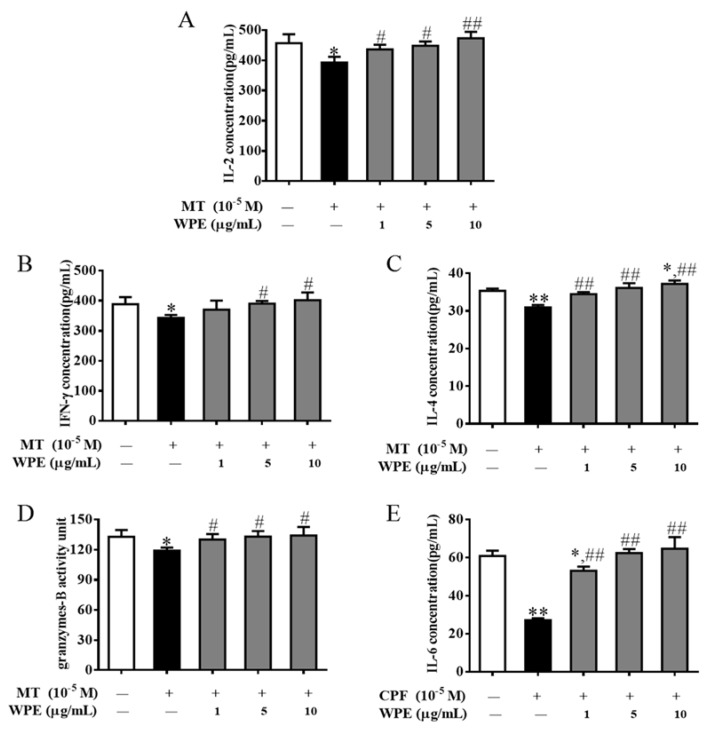
Effect of WPE on cytokine/granzyme production in splenocytes exposed to MT or CPF. Levels of (**A**) IL-2, (**B**) IFN-γ, (**C**) IL-4, (**D**) granzyme B, and (**E**) IL-6 released into culture media were then measured by ELISA. Results are presented as mean ± SD of three separate experiments. * *p* < 0.05 or ** *p* < 0.01 vs. untreated control. ^#^ *p* < 0.05 or ^##^ *p* < 0.01 vs. MT or CPF treatment.

WPE (1 µg/mL) increased the synthesis of IL-2, IFN-γ, IL-4, and granzyme B from 86.0% to 95.5% (392.9 to 436.4 pg/mL), 88.4% to 95.4% (343.1 to 370.4 pg/mL), 87.5% to 97.4% (31.0 to 34.5 pg/mL), and 89.7% to 98.0% (119.0 to 130.1 units), respectively, in T cells exposed to MT relative to the control (Figure 6A–D). In addition, WPE (1 µg/mL) significantly increased the production of IL-6 by B cells exposed to CPF from 44.6% to 87.1% (27.1 to 53.0 pg/mL) relative to the control (Figure 6E). Therefore, WPE prevents the MT- or CPF-induced decrease in the production of T- or B-cell-associated cytokines/granzyme by splenic lymphocytes.

### 3.4. Effects of WPE on Levels of Apoptosis-Associated Proteins

**Figure 7:**
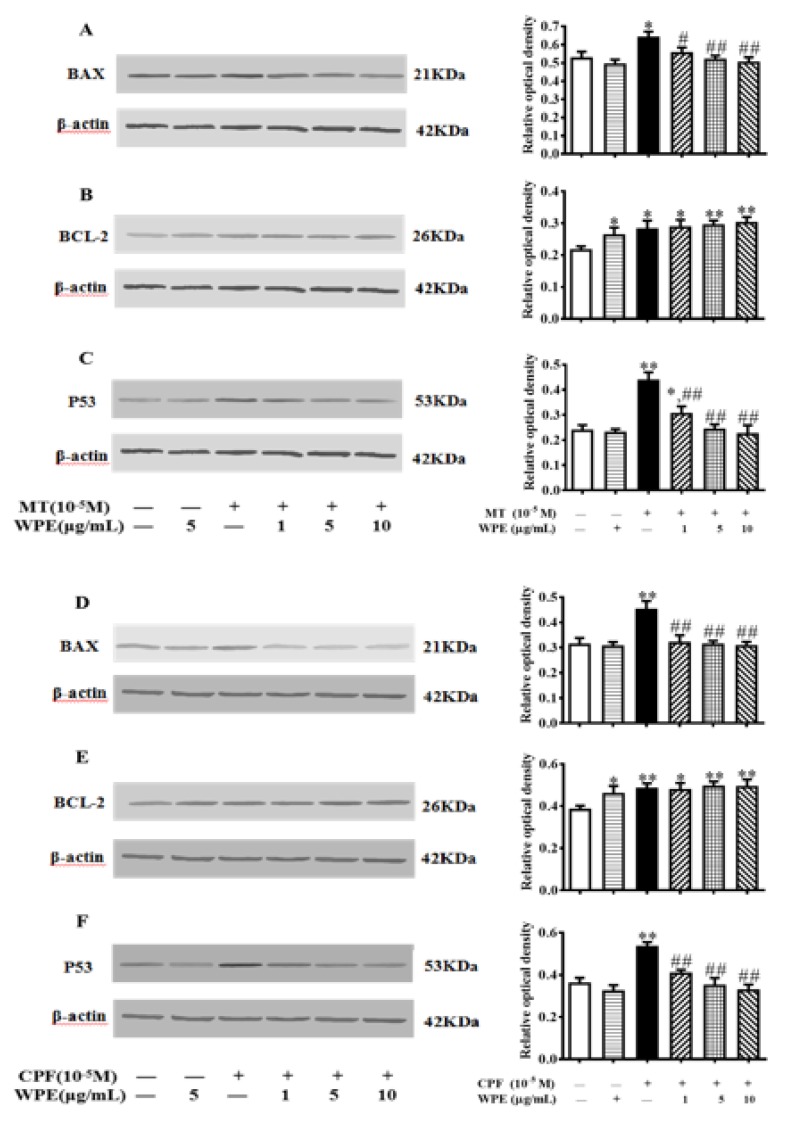
Effects of WPE on the expression of apoptosis-associated proteins in MT-exposed T cells and CPF-exposed B cells. The expression of (**A**) Bax, (**B**) Bcl-2, and (**C**) p53 in splenic T cells and (**D**) Bax, (**E**) Bcl-2, and (**F**) p53 in splenic B cells was measured using Western blotting. Results are presented as mean ± SD of three separate experiments. * *p* < 0.05 or ** *p* < 0.01 vs. untreated control; ^#^ *p* < 0.05 or ^##^ *p* < 0.01 vs. MT or CPF treatment.

Next we investigated the effects of WPE on levels of apoptosis-associated proteins (Bax, Bcl-2, and p53) with Western blotting. Levels of Bax, Bcl-2, and p53 were significantly increased in MT-exposed T cells and in CPF-exposed B cells. WPE significantly decreased Bax and p53 levels in T cells stimulated by MT and in B cells exposed to CPF (Figure 7A,C,D,F). However, WPE did not significantly affect the expression of Bcl-2 in cells exposed to MT or CPF (Figure 7B,E). Therefore, WPE suppresses the MT- and CPF-induced apoptosis of splenic lymphocytes.

### 3.5. Effects of WPE on the Production of ROS in MT- and CPF-Stimulated Cells

**Figure 8:**
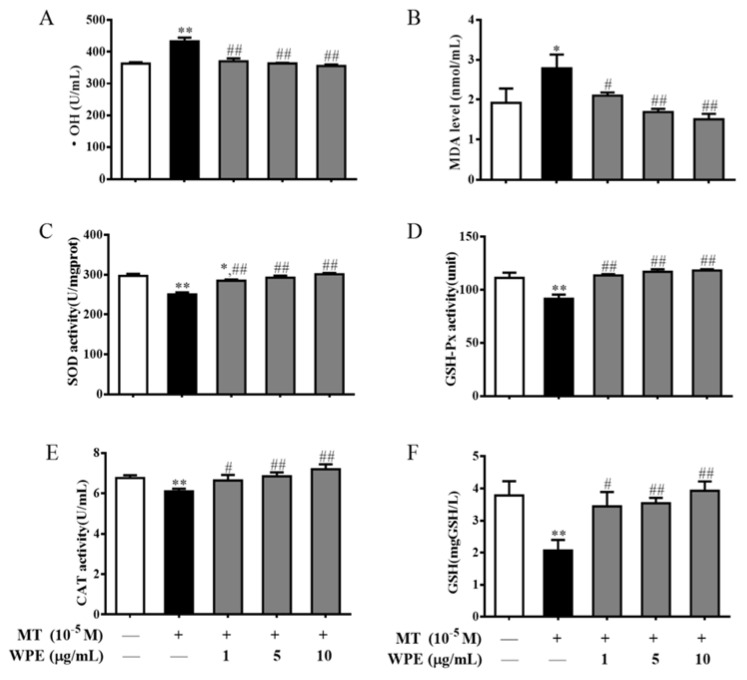
Effects of WPE on the oxidative stress parameters in splenic T cells exposed to MT. Changes in (**A**) • OH content, (**B**) MDA content, (**C**) SOD activity, (**D**) GSH-Px activity, (**E**) CAT activity, and (**F**) GSH content in T cells were measured using specific assay kits. Results are presented as mean ± SD of three separate experiments. * *p* < 0.05 or ** *p* < 0.01 vs. untreated control; ^#^ *p* < 0.05 or ^##^ *p* < 0.01 vs. MT treatment.

**Figure 9:**
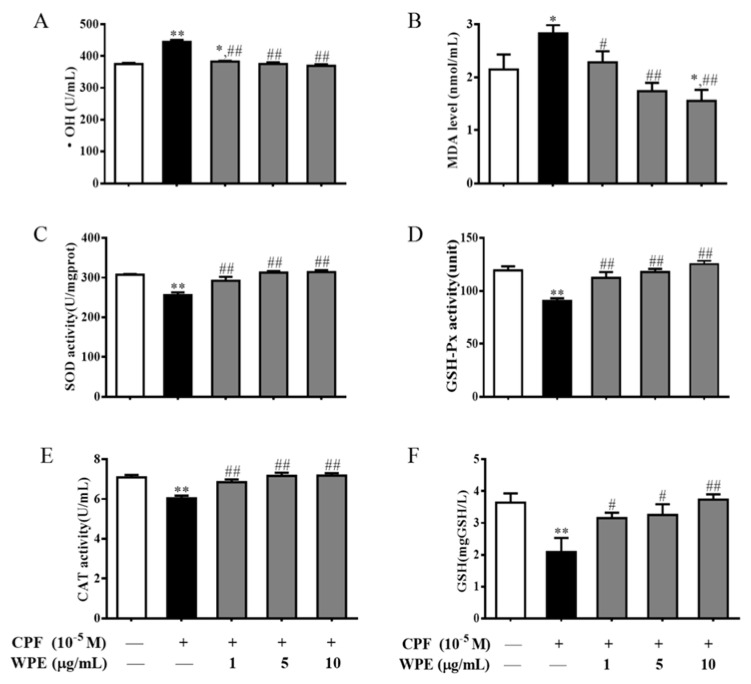
Effects of WPE on the oxidative stress parameters in splenic B cells exposed to CPF. Changes in (**A**) • OH content, (**B**) MDA content, (**C**) SOD activity, (**D**) GSH-Px activity, (**E**) CAT activity, and (**F**) GSH content in B cells were measured using specific assay kits. Results are presented as mean ± SD of three separate experiments. * *p* < 0.05 or ** *p* < 0.01 vs. untreated control; ^#^ *p* < 0.05 or ^##^ *p* < 0.01 vs. MT treatment.

Next we evaluated levels of •OH, MDA, SOD, GSH-Px, CAT, and GSH in MT- and CPF-exposed splenic lymphocytes. As shown in Figure 8, MT significantly increased •OH and MDA levels and markedly decreased SOD, GSH-Px, and CAT activity and GSH content in splenic T cells. CPF markedly increased •OH and MDA levels and significantly decreased SOD, GSH-Px, and CAT activity and GSH content in splenic B cells (Figure 9). Therefore, MT and CPF induce overproduction of ROS and oxidative stress in splenic lymphocytes. 

However, the effects of MT were significantly attenuated by WPE at 1 µg/mL; •OH and MDA levels decreased from 119.4% to 102.0% (432.9 to 369.9 U/mL) and from 145.2% to 109.5% (2.8 to 2.1 nmol/mL), respectively, relative to the controls (Figure 8A,B). By contrast, WPE at 1 µg/mL significantly increased the activity of SOD, GSH-Px, and CAT from 84.3% to 95.9% (250.5 to 285.0 U/mg prot), 82.3% to 102.1% (91.6 to 113.6 units), and 90.2% to 98.2% (6.1 to 6.6 U/mL), respectively, and GSH content from 54.6% to 91.0% (2.1 to 3.4 mg/L) relative to the controls (Figure 8C–F). WPE at 1 µg/mL also significantly decreased levels of •OH and MDA from 118.7% to 102.1% (444.6 to 382.6 U/mL) and from 131.9% to 106.4% (2.8 to 2.3 nmol/mL) in splenic B cells exposed to CPF; it also significantly upregulated the activity of SOD, GSH-Px, and CAT from 83.2% to 95.1% (255.9 to 292.4 U/mg prot), 75.8% to 94.1% (90.5 to 112.4 units), and 85.1% to 96.6% (6.0 to 6.9 U/mL), respectively, and GSH content from 57.3% to 86.7% (2.1 to 3.2 mg/L) relative to the controls (Figure 9). Therefore, WPE inhibits MT- and CPF-mediated overproduction of ROS and oxidative stress in splenic lymphocytes.

### 3.6. Effects of WPE on MT- or CPF-Induced NOX and TLR Expression

**Figure 10:**
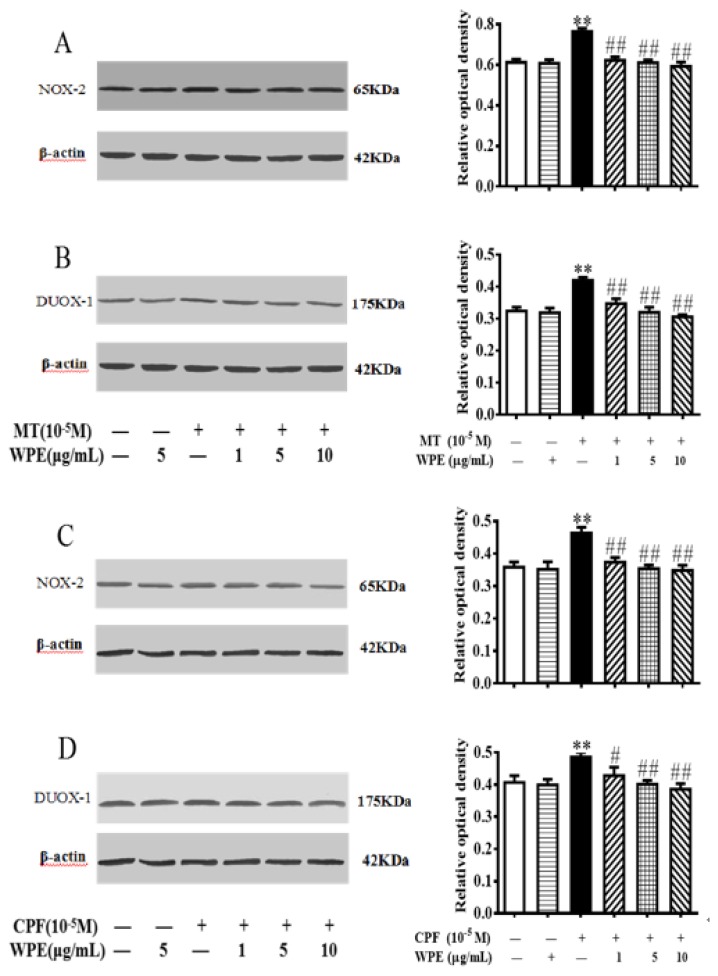
Effect of WPE on the expression of NADPH oxidase in splenic lymphocytes exposed to MT or CPF. The expression of (**A**) NADPH oxidase 2 (NOX2) and (**B**) dual oxidase 1 (DUOX1) in splenic T cells; and (**C**) NOX2 and (**D**) DUOX1 in splenic B cells was measured using Western blotting. Results are presented as mean ± SD of three separate experiments. * *p* < 0.05 or ** *p* < 0.01 vs. untreated control; ^#^ *p* < 0.05 or ^##^ *p* < 0.01 vs. MT or CPF treatment.

To determine whether WPE inhibits the overproduction of ROS through NADPH oxidase complexes (NOX), we evaluated the expression of NOX2 and DUOX1 in splenic lymphocytes treated with MT or CPF alone or in combination with WPE. As shown in Figure 10, NOX2 and DUOX1 expression was significantly increased in MT- and CPF-stimulated cells. The NOX2 and DUOX1 overexpression induced by MT or CPF was significantly reduced by WPE; at >1 µg/mL, the effects of WPE were nonsignificant in a concentration-dependent manner (Figure 10).

**Figure 11:**
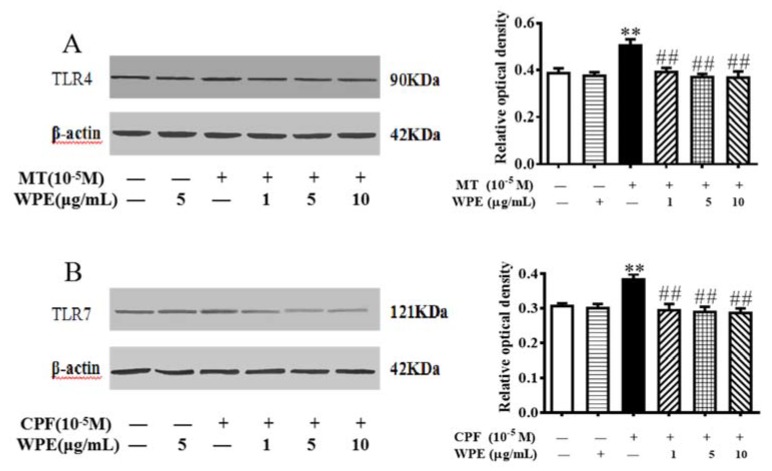
Effect of WPE on the expression of toll-like receptor (TLR)x in MT- or CPF-stimulated splenic lymphocytes. The expression of (**A**) TLR4 in splenic T cells and (**B**) TLR7 in splenic B cells was measured using Western blotting. Results are presented as mean ± SD of three separate experiments. * *p* < 0.05 or ** *p* < 0.01 vs. untreated control; ^#^ *p* < 0.05 or ^##^ *p* < 0.01 vs. MT or CPF treatment.

The expression of TLRs is critical for the production of ROS following NADPH oxidase activation [33,34,35]. TLR4 is expressed in T cells [34], and TLR7 is expressed in B cells [36]. As shown in Figure 11, MT markedly increased the expression of TLR4 in T cells, and CPF markedly increased the expression of TLR7 in B cells. Therefore, MT- or CPF-induced overactivation of NOX2 and DUOX1 might be due to overexpression of TLR4 or TLR7 in splenic lymphocytes. However, overexpression of TLR4 or TLR7 was normalized by WPE in cells treated with MT or CPF (Figure 11). Therefore, WPE inhibits overactivation of NOX2 and DUOX1 by suppressing MT- and CPF-induced overexpression of TLR4 and TLR7 in T and B cells, respectively. 

## 4. Discussion

We investigated the effects of WPE on MT- and CPF-induced immunotoxicity using mouse splenocytes. The major findings were as follows: First, WPE attenuated the cytotoxicity of MT and CPF by increasing cell viability and recovering splenic lymphocyte subpopulations. Second, WPE significantly restored the production of IL-2, IL-4, IL-6, IFN-γ, and granzyme B in splenic lymphocytes exposed to MT and CPF. Third, WPE prevented MT- and CPF-induced oxidative damage by normalizing levels of GSH, GSH-PX, SOD, CAT, •OH, and MDA. Finally, WPE inhibited NOX2 and DUOX1 overexpression by suppressing the expression of TLR4 and TLR7. Therefore, WPE protects against MT- and CPF-mediated immunotoxicity by modulating TLRx-NOX-ROS.

The immunotoxicity of OPs has been reported [6,7,37,38]. The spleen is an important immune organ and harbors mainly T and B lymphocytes, which are critical for immunity in animals and humans [39]. OPs, such as diazinon, methyl parathion, and fenitrothion, have toxic effects on the spleen and splenic lymphocytes [30,37]. Polyphenols reportedly protect against the toxicity of OPs [40,41]. Curcumin increases the proliferation of blood lymphocytes exposed to parathion [41], and walnut polyphenols regulate proportions of murine splenic T-cell subpopulations in response to fenitrothion [28]. In this study, MT and CPF significantly suppressed the proliferation of T and B cells, which was significantly reversed by WPE.

Immune-related cytokines play important roles in activating and modulating immune responses and thus are useful for evaluating immune function [21,42]. However, the pesticide pirimiphos-methyl significantly decreases the production of IL-2, IL-4, IL-6, and IFN-γ in mouse spleen [43]; dimethoate significantly decreases synthesis of IL-2, IL-4, and IFN-γ in mouse spleen [44]; and diazinon decreases IL-2, IL-4, IL-10, IL-12, and IFN-γ levels in splenocytes [45]. Polyphenols have profound effects on cytokine secretion [21,23]. In vivo, curcumin increases levels of IFN-γ and IL-1β in Th1 cells, counteracting nicotine-induced toxicity in rats [20]. Similarly, lychee fruit polyphenols decrease the secretion of IL-6 and TNF-α, thus having anti-inflammatory effects in peripheral-blood monocytes [23]. In this study, MT reduced levels of T-cell-related cytokines, and CPF reduced B-cell-related cytokine, in splenocytes. WPE increased the production of T-cell-related cytokines in splenocytes exposed to MT and the production of B-cell-related cytokine in those exposed to CPF, consistent with the MTT assay and flow cytometry results.

Exposure to OPs can result in the overproduction of ROS, leading to oxidative stress [46,47,48]. Diazinon influences the activity of antioxidant enzymes, causing severe damage to macrophages [48]. Pirimiphos-methyl increases the overproduction of ROS in mice [46]. Polyphenols have antioxidant effects, protecting against damage caused by toxic chemicals [17,28,49]. Pomegranate polyphenols decrease the ROS generation and oxidative damage induced by arsenic [50]. WPE is rich in phenolic compounds, such as ellagitannins and flavonoids. The content of ellagic acid and ellagic tannin, the main components of WPE, is 86.54% [28]. Ellagitannins have radical-scavenging activity due to their phenolic hydroxyl groups [51]. Moreover, flavonoids inhibit free-radical chain reactions by acting as chelators of transition metals via their •OH groups [52]. In this study, MT and CPF induced overproduction of ROS and oxidative stress in splenic lymphocytes, as indicated by significant increases in •OH and MDA levels and marked decreases in SOD, GSH-Px, and CAT activity and GSH content. WPE protected against MT- and CPF-induced oxidative damage by maintaining or increasing the activity of several key antioxidants and decreasing levels of •OH and MDA in splenic lymphocytes.

OPs cause apoptosis of diverse cell types by inducing excessive ROS production. Bax (pro-apoptotic), Bcl-2 (anti-apoptotic), and p53 (pro-apoptotic) play important roles in apoptosis [53,54]. Avermectin induces apoptosis by upregulating the Bax/Bcl-2 ratio and mediating the overproduction of ROS in HepG2 cells [55]. Paraoxon and malaoxon induce apoptosis in human pulmonary cells by inducing oxidative stress [56]. Polyphenols prevent apoptosis by altering the expression of apoptosis-related proteins [57]. Strawberry polyphenols suppress the apoptosis of HepG2 cells by decreasing the intracellular ROS level [58]. Kaempferol upregulates the expression of Bcl-xL and downregulates that of p53 and Bax by inhibiting extracellular regulated protein kinases (ERK), NF-κB, and MyD88 expression in human ovarian cancer cells [59]. In this study, MT and CPF induced apoptosis of splenic lymphocytes by significantly increasing Bax and p53 levels, and WPE prevented apoptosis by restoring the expression of Bax and p53.

NADPH oxidase mediates ROS biosynthesis [60] and can be present in inactivated, startup, activated, and hyperactivated states [61]. Exposure to toxins, including organic environmental pollutants, results in hyperactivation of NADPH oxidase, increasing ROS generation [62]. NADPH oxidase is a holoenzyme comprising two membrane-bound subunits (gp91phox and p22phox) and cytosolic regulatory components (Rac, p67phox, p40phox, and p47phox) [9]. Gp91phox is the major catalytic subunit of NADPH oxidase. The NADPH oxidase homologues NOX1, NOX2, NOX3, NOX4, NOX5, DUOX1, and DUOX2 are expressed in a variety of cell types [9,10]. NOX2 and DUOX1 are expressed in T and B lymphocytes [28]. Fenitrothion increases the expression of NOX2 and DUOX1 in splenic T cells [28]. NOX2 is activated by paraquat in innate immune cells [10,63]. Polyphenols inhibit the expression of NADPH oxidase, balancing the ROS level and maintaining homeostasis upon stimulation [52,64,65]. Flavonoids suppress the expression of NADPH oxidase (DUOX1) [52]. Strawberry polyphenols reduce excessive ROS production by activating NOX2 [65]. Here MT and CPF induced overexpression of NOX2 and DUOX1 in splenic lymphocytes, which was significantly restored by WPE.

TLRs play an important role in immunity via their link with NADPH oxidase homologues [33]. Stimulant recognition by TLRs stimulates NADPH oxidase-mediated ROS formation, an important component of cellular regulation [28]. The expression of TLRs is critical for the production of ROS following NADPH oxidase activation [33,34,35]. TLR4 is a pathogen-associated molecular pattern receptor expressed in phagocytes and T cells [66]. NOX2 is activated by the overexpression of TLR4, a key regulator of innate immunity [34]. LPS-induced activation of TLR4 in vitro enhances generation of ROS by NOX2 in T cells [35]. In addition, TLR4 ligands increase NOX2 expression in dendritic cells [45]. TLR7 is present and functional in B lymphocytes, in which it regulates NADPH oxidase expression and ROS production [47]. TLR7 ligands trigger NADPH oxidase activation, resulting in a high level of ROS in B cells [47]. Influenza A virus infection increases NOX2 oxidase-dependent oxidative stress by upregulating the expression of TLR7 [67]. However, polyphenols decrease the overexpression of TLRs and normalize the level of NADPH oxidase [28,35]. Polyphenols from *Antirhea borbonica* decrease NOX2-mediated ROS generation in LPS-exposed adipocytes by reducing the expression of TLRs [68]. Similarly, curcumin suppresses LPS-induced, NOX-mediated ROS production by inhibiting TLR4 signaling in rat vascular smooth muscle cells [35]. In this study, MT and CPF caused overexpression of TLR4 and TLR7 in T and B cells, respectively. WPE decreased overexpression of TLR4 in T cells and overexpression of TLR7 in B cells, which suggests that its normalization of NOX2 and DUOX1 was mediated by the regulation of TLR4 in splenic T cells and TLR7 in splenic B cells.

## 5. Conclusions

MT and CPF affect the proliferation of, and cytokine/granzyme production by, splenic T and B lymphocytes, respectively, in vitro. WPE protects against MT- and CPF-induced cytotoxicity by inhibiting their effects on cell viability and cytokine/granzyme production in vitro. The protective effects of WPE in MT- or CPF-exposed splenic T and B cells are mediated by the inhibition of oxidative stress via suppression of the activation of NOX2 and DUOX1, the latter caused by downregulation of TLR4 and TLR7, respectively. Further studies are needed to identify the mechanisms underlying the effects of MT and CPF on splenic T and B lymphocytes and the protective effects of WPE.
